# Supplementary material for: Prevalence of arps10, fd, pfmdr-2, pfcrt and pfkelch13 gene mutations in Plasmodium falciparum parasite population in Uganda
Source: PLoS One. 2022 May 5;17(5):e0268095. doi: 10.1371/journal.pone.0268095 (PMC9070901; doi:10.1371/journal.pone.0268095)
Supplement: S1 Table — (DOC) [file pone.0268095.s001.doc]

**SUPPORTING INFORMATION**

**S1 table: PCR cycling conditions for amplification of *P. falciparum* DNA fragments sandwiching *fcrt* N326S, *fd* D193Y, *arps10* V127M and *mdr2* T484L genetic background mutations**

| **Gene** | **Mutation** | **First round PCR** | **Second round PCR** |
| --- | --- | --- | --- |
| *fd* | D193Y | Enzyme activation and initial denaturation at 95°C for 15 minutes followed 55 cycles of denaturation at 94°c for 30s , annealing at 57°C for 90 sec, extension for 90sec and final extension for 10 minutes | Not performed |
| *arps10* | V127M | Enzyme activation and initial denaturation at 95°C for 15 minutes followed 55cycle of denaturation at 94°c for 30s , annealing at 57°C for 90 sec , extension for 90sec and final extension for 10 minutes | Not performed |
| *mdr2* | T484L | Enzyme activation and initial denaturation at 95°C for 15 minutes followed 55cycle of denaturation at 94°c for 30s , annealing at 57°C for 90 sec , extension for 90sec and final extension for 10 minutes | Enzyme activation and initial denaturation at 95°C for 15 minutes followed 55 cycles of denaturation at 94°c for 30s , annealing at 57°C for 90 sec , extension for 90sec and final extension for 10 minutes |
| *crt* | N326S | Enzyme activation and initial denaturation at 95°C for 15 minutes followed 55cycle of denaturation at 94°c for 30s , annealing at 57°C for 90 sec , extension for 90sec and final extension for 10 minutes | Enzyme activation and initial denaturation at 95°C for 15 minutes followed 55cycle of denaturation at 94°c for 30s , annealing at 57°C for 90 sec , extension for 90sec and final extension for 10 minutes |
