# Supplementary material for: Prevalence of arps10, fd, pfmdr-2, pfcrt and pfkelch13 gene mutations in Plasmodium falciparum parasite population in Uganda
Source: PLoS One. 2022 May 5;17(5):e0268095. doi: 10.1371/journal.pone.0268095 (PMC9070901; doi:10.1371/journal.pone.0268095)
Supplement: S2 Table — (DOC) [file pone.0268095.s002.doc]

**SUPPORTING INFORMATION**

**S2 table: Primer sets used during amplification of *Plasmodium falciparum*** DNA

| **Gene** | **Mutation** | **First round PCR primers** | **Second round PCR primers** |
| --- | --- | --- | --- |
| *fd (*ferredoxin) | fd D193Y | PFFD-F1 gctcattccccatttcaatcatatcca  PFFD-R1 agttgttcttacatgcgcagc | Not applicable |
| *arps10* (apicoplast ribosomal protein S10) | V127M | Pf arps-F tgcgacttttagggtgtggaa  Pf arps-R acccacaattctgggtaatttca | Not applicable |
| *mdr2* (multidrug resistance protein 2) | T484L | Pfmdr - F1catatttgtggccaagcaaaag  Pfmdr-R1 cattatccatctcatttg | Pfmdr-F2 ggttgggcatcaaatgaag Pfmdr-R1 cattatccatctcatttg |
| *crt* (chloroquine resistance transporter) | N326S | FCRT-F1 gaagaaacacagtcgtagaggta  FCRT-R1 ggtctcttacaacatcacccta | FCRT-F2 gggtaaccatataatgttgcat  FCRT-R2 ggttctcttacaacatcacccta |
| K13 | - | K13-1 5′-cggagtgaccaaatctggga-3′ and K13-4 5′-gggaatctggtggtaacagc-3′) | nested PCR (K13-2 5′-gccaagctgccattcatttg-3′ and  K13-3 5′-gccttgttgaaagaagcaga-3′) |
