# Supplementary material for: Prevalence of arps10, fd, pfmdr-2, pfcrt and pfkelch13 gene mutations in Plasmodium falciparum parasite population in Uganda
Source: PLoS One. 2022 May 5;17(5):e0268095. doi: 10.1371/journal.pone.0268095 (PMC9070901; doi:10.1371/journal.pone.0268095)
Supplement: Attachment — Submitted filename: Response to Reviewers.doc [file pone.0268095.s003.doc]

**Response to Reviewers Comments on manuscript PONE-D-21-33254**

 The current manuscript was previously submitted with the unique identifier, PONE-D-21-08961. The manuscript was reviewed and we were requested to resubmit. We have now addressed all the comments raised in the previous revisions and here below is a summary of our point-by-point response.

**General comments: Reviewer #1:** The author should be congratulated for conducting an imperative study. This is a very relevant research topic that adds to the body of knowledge on artemisinin resistance. The paper is generally very well written and structured. However, it has minor shortcomings in regards to gramma, spacing and some background information is lacking.

**Response:** Thanks for the comment, corrections to the grammatical errors have been effected in the revised manuscript. Additional information has been incorporated in the background of the revised manuscript.

**Reviewer #1: Abstract, K13 should be written in full when mentioned the first time**

**Response:** This has been revised, *Plasmodium falciparum* Kelch 13 *(PfKelch13)* gene.

………..…………………………………………………………………………………. *page 2, line 32*

**Reviewer #1: The keys should not include the same word that appear in the title.**

**Response:** This has been revised and the following terms have been incorporated.

*Resistance development,* *molecular drivers, genetic background, artemisinin resistance*

………………………………………………………………………………page 3, line 55, 56

**Reviewer #1:** A basic description of genetic background should be included in the background information

**Response:** Unique parasite genetic background support independent emergence of K13 mutations associated with artemisinin resistance in different geographical areas in Southeast Asia (Takala-Harrison *et al.,* 2014). A study by Miotto *et al.,* 2015 reported a strong association between nonsynonymous mutations in *fd* (ferredoxin), *arps10* (apicoplast ribosomal protein S10), *mdr-2* (multidrug resistance protein 2) and *crt* (chloroquine resistance transporter) genes and development of artemisinin resistance in *P. falciparum* parasite. The background mutations shown to be associated with independent development of artemisinin resistance in Southeast Asia include, *fd-*D193Y, *aps10-*V127M, *mdr2-*T484I, *crt-*I356T and *crt-*N326S (Miotto *et al.,* 2015). This finding has helped define the role of genetic background in driving independent development and spread of artemisinin resistance in Southeast Asia. Several K13 mutations whose role in development of artemisinin resistance is not known have been reported in sub-Saharan Africa. With the likely threat of emergence of artemisinin resistance in sub-Saharan Africa understanding the drivers of artemisinin resistance development is critical in guiding establishment of effective control strategies to combat resistance.

………………………………………………………………………………page 4,5; line 82-91

**Reviewer #1:** Material and methods: A brief description of the original study could be included

**Response:** Thanks for the comment. We had mentioned primary studies from which we obtained samples for our study however, further description of these studies has been added in the revised manuscript.

We used samples from two previous studies conducted in Uganda; a randomized, single-blinded, longitudinal clinical trial designed to compare safety, tolerability and efficacy of three different combination antimalarial regimens for treatment of uncomplicated malaria done from November 2004 to June 2006 in Kampala (Dorsey *et al.,* 2007); and a cohort study, Program for Resistance, Immunology, Surveillance and Modeling of Malaria in Uganda (PRISM 2) conducted in three districts Jinja, Kanungu and Tororo from August 2011 to December 2018 (Kamya *et al.,* 2020). In both studies malaria parasite infected blood samples were collected from children aged 1-10 years (Dorsey *et al.,* 2007) and 6 months-10 years (Kamya *et al.,* 2020). Using simple random sampling, 100 samples each from the two studies were selected for inclusion in our study.

……………………………………………………………………………...page 6, line 119-134

**Reviewer #1:** The authors should consider including a map showing the sites where samples were collected to show the representativeness.

**Response:** Thanks for the comment. The map showing the sites were the samples for the primary studies were collected from can be found in a publication by Kamya *et al.,* 2020. This study is referenced in the methods section of our manuscript. The primary studies were conducted in Kampala, Jinja, Kanungu and Tororo districts in Uganda.

……………………………………………………………………………………..page 6, line 119-134

**Reviewer #1:** Line 99, the RDT name/names that were used are not mentioned

**Response:** Thanks for the comment, this has been included in the revised manuscript. In our study, we used *Plasmodium falciparum* histidine rich protein-2 (*PfHRP-2*) malaria rapid diagnostic test (mRDT) to screen the blood samples for inclusion into our study.

………………………………………………………………………………...page 6, line 137-8

**Reviewer #1:** Line 106, thin smears were mentioned, it is not clear what they were used for

**Response:** Thanks for the comment, this correction has been effected in the revised manauscript.

In our study blood samples to be included were screened for presence of *P. falciparum* parasite infection using two methods, mRDT and microscopy. We used *PfHRP-2* mRDT to screen for presence of *P. falciparum* parasites in the blood samples. For blood samples which were positive on *PfHRP-2* test a thick smear stained with 10% Giemsa was prepared and assessed using microscopy to establish presence of malaria parasites. A thin smear stained was prepared to confirm presence of *P. falciparum* parasites in the blood sample.

……………………………………………………………………………..page 7, line 139-143

**Reviewer #1:** In Line 111, 118 and for some the reagents and equipment, the details are not complete

**Response:** This has been corrected in the revised manuscript.

………………………………………………………………………page 8, line 158 and 171

**Reviewer #1:** In line 119, the authors do not mention the name of the machine used.

**Response:** A thermocycler was used in DNA amplification.

………………………………………………………………………page 8, line 157

**Reviewer #1:** Line 124, ACTG is not given in full.

**Response:** The samples were sequenced at ACGT Inc. (Wheeling IL, USA) facility. The letters in ACGT stand for the four nucleotides of the DNA, Adenine Cytosine Guanine and Thymine. This has been provided in the revised manuscript.

……………………………………………………………………page 8, line 161-2, 174

**Reviewer #1:** The presentation of the results can be confusing to the readers (line 152-156), this could be recast

**Response:** Thanks for the comment, this section of the results has been reorganized in the revised manuscript.

……………………………………………………………………page 9-10, lines 189-127

**Reviewer #1:** Line 177, has a grammatical error.

**Response:** This has been corrected in the revised manuscript.

**Reviewer #1:** Discussion; Line 218 has a grammatical error.

**Response:** This has been corrected in the revised manuscript.

**Reviewer #1:** References: The references list requires some attention, reference no. 4 and 15 are both WHO document, could they be written properly

**Response:** This has been effected in the revised manuscript.

- World Health Organization. World Health Organization: 20 years of global progress and challenges. Geneva, Switzerland: World Health Organization; 2020. <https://www.who.int/publications/i/item/9789240015791>, Accessed 20^th^ July 2021

**…………………………………………………………………**page 15, line 318-320

- World Health Organization. Basic malaria microscopy: Part I. Learner's guide: Part II. Tutor's guide (2nd ed.). Malaysia WHO; 2004. <http://apps.who.int/iris/bitstream/handle/10665/44208/9789241547918_eng.pdf;jsessionid=9231B8D7E40D189B944F3693C1C140E8?sequence=2>, Accessed 20^th^ July 2021

……………………………………………………………………...page 16, line 370-373

**Reviewer #1:** References: Names in the reference list e.g P.falcuparum are not in italics

**Response:** This has been corrected in the revised manuscript.

……………………………………………………………………...page 15, 16; line 326-399

**Reviewer #2:** The topic of the paper is important in the field as this is work on K13 and antimalaria drug resistance in Uganda as endemic for malaria where first record of delayed artemisinin parasite clearance phenotype was documented there. The idea is sound. However, there is a major issue in this paper is the date of sample collection is in earlier days in 2004 this is not good point for monitoring the K13 gene mutations which appeared after introduction of ACTs. Unless the author wants to compare the results with recent collected samples e.g 2016 or 2020 but this not the case. Also the statistical analysis is poor mainly descriptive and missing. Also there is no information in the complexity of infection (COI) and parasite species identification by PCR and no data on mixed infection

**Response:** Thanks for the comment. Our study intended to assess the genetic background of *P. falciparum* parasites in Uganda and their association with K13 mutations. This is critical as a study by Miotto *et al.,* 2015 showed that K13 mutations associated with artemisinin resistance independently arise in malaria parasites with unique genetic background. We therefore, intended in our study to screen and compare the prevalence of genetic background and K13 mutations in *P. falciparum* before and after the introduction of artemisinin agents in Uganda. Use of Artemisinin based therapies in malaria treatment was rolled out in Uganda in 2006 (Nanyunja, 2011).

Due to the few mutations which were found in the study, the analysis was restricted mainly to descriptive. More information has however been added from further analysis of the results. For the background mutation and K13 mutations found in our study, additional information from further analysis has been incorporated in the revised manuscript. Information on year of sample collection has been provided for those samples that had the mutations. The samples for our study were obtained from two previous studies Dorsey *et al.,* 2007 (November 2004 to June 2006) and Kamya *et al.,* 2020 (August 2011 to December 2018). Using simple random sampling, 100 samples were selected from each of the two studies. Of the 200 samples analyzed 142 were successfully sequenced, 51 (2005 samples) and 91 (2015 samples). For samples collected in 2005 prior to the introduction of ACTs in malaria treatment in Uganda one K13 nonsynonymous K13 mutation, Y588N and one synonymous SNP, I587I were found. Analysis of samples collected in 2015 found five nonsynonymous K13 mutations E643K, E596V, S600C and A578S.

…………………………………………………………………………….……………...page 10, line 208-213

Our study was based on samples collected from two previous studies where information on details on the complexity of infection and malaria parasite species were reported in the articles by Dorsey *et al.,* 2007 and Kamya *et al.,* 2020. Our current study intended to screen for genetic background and K13 mutations present in the *P. falciparum* parasites found in selected samples from the two studies. However, the sequences were analyzed using Mutation Surveyor (SoftGenetics LLC., version 5.1, State College, PA, USA) in order to determine mixed alleles based on the presence of two chromatogram peaks at one nucleotide site, and to also reduce the presence of false positive and (or) false negative mutant sites (Huang *et al.* 2021, Aldakeel et al., 2020). The blood samples were screened for presence of *P. falciparum* parasites using *PfHRP-2* mRDT and microscopy. We did not perform PCR to further confirm the species of the parasites, we used *PfHRP-2* mRDT which is specific for *P. falciparum* parasites and thin blood smear stained with 10% Giemsa for *P. falciparum* species identification.

……………………………………………………page 6, lines 116-130 and lines 131-144

**Reviewer #2:** Abstract: background is week with no updated data on epidemiological profile of drug resistance and malaria in Uganda.

**Response:** Thanks for the comment. The last paragraph in the introduction section of our manuscript provides recent information on status of K13 mutations in Uganda and neighboring countries. We have also provided further information on the genetic background in the introduction section of the revised manuscript. Due to the word limitation in the abstract, additional requested information has been provided in the introduction section to the revised manuscript.

…………………………………………………….page 3, 5; lines 70-74 and lines 105-106

**Reviewer #2:** The introduction is not written in properly. There is no information regarding the epidemiological profile of malaria in Uganda, the prevalence and species estimates and status of drug resistance and current drugs used currently all are missing information.

…………………………………………………….page 3, 5; lines 70-75 and lines 105-106

**Response:** This information has been provided in the revised manuscript.

Uganda is among the 29 malaria endemic countries which contributed 95% of malaria cases globally in 2019 (WHO malaria report, 2020). Artemether-lumefantrin (AL) and injectable artesunate are the first-line agents for uncomplicated and severe malaria respectively. Artemisinin agents remain highly effective in the treatment of malaria in Uganda (Rasmussen *et al.,* 2017). *Plasmodium falciparum* is responsible for over 99% of all malaria infections in Uganda (WHO world malaria report, 2020, Asua *et al.,* 2017).

……………………………page 2 line 30-31, page 3, lines 70 -72; page 5, lines 105-106

**Reviewer #2:** Materials and methods is good but missing the accurate numbers of samples in which year and also the accession numbers of Sanger sequences obtained not indicated so authors need to declare these an open repository or database.

**Response:** The samples for our study were obtained from previous studies in Uganda. 100 samples each were obtained from, a single blind clinical trial done between November 2004 and June 2006 (Dorsey *et al.,* 2007) and a cohort study (PRISM 2) done from August 2011 to December 2018 (Kamya *et al.,* 2020). In both studies malaria parasite infected blood samples were collected from children aged 1-10 years (Dorsey *et al.,* 2007) and 6 months-10 years (Kamya *et al.,* 2020).

We deposited sequences for K13 mutations detected in our study (A578S, Y588N, E596V, E643K, S600C, W565X and I587I) and two sequences both for *mdr-2* gene mutations (*ile*492*Val, Tyr*423*Phe*) and *arps10* gene mutations (*Val*127*Met*, *Asp*128*His*) in NCBI data base*.* The sequence GenBank accession number MZ668587-MZ668597). During processing of sequences for depositing in NCBI database, sequence data was re-analyzed using Mutation Surveyor (SoftGenetics LLC., version 5.1, State College, PA, USA) in order to determine mixed alleles based on the presence of two chromatogram peaks at one nucleotide site, and to also reduce the presence of false positive and (or) false negative mutant sites (Huang *et al.* 2021, Aldakeel et al., 2020). Fasta file were aligned using UGENE v.39 (Unipro) to references PF3D7_1343700 (*Pfkelch13*), PF3D7_1460900.1 (*arps10*), PF3D7_1318100 (*fd*), PF3D7_1447900 (*mdr-2*) and PF3D7_070900 (*crt*) (Huang *et al.* 2021, Aldakeel et al., 2020).

From the re-analysis, mutations F446I, F483L, F491F, M660M, A661S were removed from the list of the reported mutations in our study as they occurred in a low quality region of the sanger traces at the ends, <20% (Huang *et al.* 2021, Aldakeel et al., 2020). Additionally, we found three new mutations, S600C, W565X and I587I which has been included in the revised manuscript. The sanger traces were deposited in the GenBank and can be accessed using the accession numbers MZ668587-MZ668597 and MZ818701-MZ818770.

………………………………………………..page 5, lines 116-130; page 9, lines 186-188

**Reviewer #2:** Results is missing the comparative analysis between different years and statistical analysis.

**Response:** The samples analyzed in our study were collected in 2005 and 2015 from previous studies by Dorsey *et al.,* 2007 and Kamya *et al.,* 2020 respectively. For *P. falciparum* parasites collected in 2005 prior to the introduction of ACTs in malaria treatment in Uganda, one nonsynonymous K13 mutation Y588N was found. Analysis of *P. falciparum* parasites collected in 2015 found five nonsynonymous K13 mutations E643K, E596V, W565X, S600C and A578S. Genetic background mutations were found in *mdr2* gene (*ile*492*Val, Tyr*423*Phe*) and *arps10* gene (*Val*127*Met, Asp*128*His*) in the *P. falciparum* parasite samples screened. Mutation in the *mdr2* gene, *Tyr*423*Phe* was found in 4, 35 *P. falciparum* parasites samples collected in 2005 and 2015 respectively while mutation *ile*492*Val* was found only in 15 parasite samples collected in 2015. The mutations in *arps10* gene *Val*127*Met* and *Asp*128*His* were each found in one *P. falciparum* parasite sample collected in 2015.

…………………………page 3, lines 116-130; page 9, lines 200-203, page 11, line 227

**Reviewer #2:** Discussion needs major re-writing following standard procedures for writing the discussion. Authors should have mentioned and discussed that major issue of this paper that the samples has been collected in 2004 so they can justify the results as no mutation observed in the genetic background marker and compare it with recent studies published

**Response:** Thanks for the comment, this has been effected in the revised manuscript.

The samples for this study were obtained from two previous studies conducted from November 2004 to June 2006 (Dorsey *et al.,* 2007) and August 2011 to December 2018 (Kamya *et al.,* 2020). For our study, 100 samples each were randomly selected from the samples collected in 2005 from a study by Dorsey *et al.,* 2007 and 2015 from a study by Kamya *et al.,* 2020. The samples analyzed in our study were collected before (2005) and after (2015) the introduction of artemisinin based therapies (ACTs) in malaria treatment in Uganda (Nanyunja, 2011). This was to help assess the role that the introduction of artemisinin based therapies for malaria treatment played in driving development of a genetic background which supports the rise of K13 mutations associated with artemisinin resistance in Uganda. Artemisinin resistance have been shown to arise independently in different settings in Southeast Asia (Miotto *et al.,* 2015 and Takala-Harrision *et al.,* 2016). This is an indicator of the role unique sets of conditions in different malaria endemic regions play in driving the development of artemisinin resistance. In our study, we detected background mutations in, *mdr2* (*ile*492*Val, Tyr*423*Phe*) and *arsp10* (*Val*127*Met, Asp*128*His*) genes in *P. falciparum* parasite samples collected in 2015 a decade after introduction of artemisinin agents in malaria treatment in Uganda (Nanyunga *et al.,* 2011). Absence of genetic background mutations in *P. falciparum* parasites collected prior (2005) to the roll out of artemisinin agents in malaria treatment points to the potential role the use of artemisinin agents play in driving development of supportive genetic background for the rise of artemisinin resistance. There is however need for further analysis of more parasite samples to help ascertain parasite genetic background in the country and its association with the development of artemisinin resistance. This is especially critical as previous studies done in Southeast Asia have demonstrated an association between parasite genetic background and the independent emergence of artemisinin resistance (Miotto *et al.,* 2014, Takala-Harrison *et al.,* 2015). A recent study in South Sudan (Hussein *et al.,* 2020) which analyzed samples collected in 2015-2017 after introduction of artemisinin agents in malaria treatment detected genetic background mutation in *Pfcrt* N326S gene which was previously reported in Southeast Asia and associated with artemisinin resistance. However, it still remains unknown whether occurrence of these background mutations in African malaria parasites has a role in driving development of artemisinin resistance.

We detected K13 SNPs in *P. falciparum* samples collected both prior (2005) and a decade (2015) after introduction of ACTs in malaria treatment in Uganda. Our findings are similar to previous studies in sub-Saharan Africa that have found diverse K13 mutations in the *P. falciparum* parasites whose role in artemisinin resistance is not known (Asua *et al.,* 2021). Despite recent reports of existence of 469Y and 675V mutations associated with artemisinin resistance in Uganda (Asua *et al.,* 2021, Ikeda *et al.,* 2018), our study did not find genetic background polymorphisms which support emergence of K13 mutations associated with artemisinin resistance previously reported in SEA (Miotto *et al.,* 2015). With no current report on artemisinin resistance in sub-Saharan Africa, understanding the factors which drive development of K13 mutations will help guide efforts towards containment and eradication of artemisinin resistance in malaria treatment.

…………………………………………………………………..…..page 11-13, lines 231-292

**Second round comments**

1. **Comments Reviewer #1**:  The manuscript has been well revised; the sciences is sound there are just minor issues observed in the write-up. The exact name and manufacturer of the mRDT used hasn’t been mentioned and the manufacturer, city and country for the reagents haven’t been written.

**Response:** The *pf*HRP-2 malaria test kits used were manufactured by, Premier Medical Corporation Ltd, Gujarat, India

2. **Comment Reviewer #3**: The authors have successfully addressed the comments raised by the reviewer previously. I am satisfied with responses provided by the authors. And therefore advise the manuscript to be accepted.

**Response:** Appreciated, thanks

3. **Comment Reviewer #4**: The authors assessed the genetic background of 200 Ugandan Pf isolates in relation to Pfk13 mutations using archived samples of Pf collected in 2005 (before the introduction of ACT in the country) and 2015.
   The introduction is pertinent and provides sufficient background information. The methods section is somewhat confusing, in particular the source(s) of PCR protocols. Sequencing showed 7 SNPs in Pfk13. The discussion section is too long for the amount of data presented in this study.
   The methodological approach is of scientific interest. However, based on 142 Pf isolates (with successful PCR-sequencing results for Pfk13), of which 7 presented Pfk13 mutations, I think that it is not possible to support the authors’ hypothesis concerning the genetic background that favors the emergence of Pfk13 mutations associated with artemisinin resistance. In addition, the authors did not perform any in vitro experiment, for example, ring stage assay (RSA), to identify Pf isolates with decreased susceptibility to artemisinin. The clinical response to ACT of the patients from whom the isolates were obtained is not known. None of the 7 SNPs found by the authors are validated or associated markers of artemisinin resistance.

**Response:** The protocols used in our study; for *pfkelch13* gene mutations a method by Ariey *et al.,* 2011 was used; for *arps10, fd, mdr-2, pfcrt* gene mutations we used nested PCR with primers developed based on a previous study by Miotto *et al.,* 2015. The discussion section is based on the key findings of the study.

The aim of the study was to assess the presence of mutations *fd-*D193Y, *aps10-*V127M, *mdr2-*T484I, *crt-*I356T, and *crt-*N326S in the *Plasmodium falciparum* parasite samples collected before and after introduction of the artemisinin based combination therapies in malaria treatment in Uganda. This is a descriptive study and we did not intend to test a hypothesis of presence or absence of a favorable parasite genetic background in Uganda.

The aim of our study was to assess for mutations in the *pfkelch13, fd, arps10, pfmdr-2* and *pfcrt* genes in the *Plasmodium falciparum* parasites before and after introduction of Artemisinin combination therapies in Uganda. Previous studies by Dorsey *et al.,* 2007 and Kamya *et al.,* 2020 from which the *P. falciparum* parasite samples for our study were obtained report results of the in vitro experiments.

The K13 mutations among *P. falciparum* parasites in sub-Saharan Africa are diverse and majority have not been validated Ocan *et al.,* 2019, Ndiwa *et al.,* 2021.

English, including punctuations, subject-verb agreement, and syntax have been corrected throughout the revised manuscript.

4. **Comment Reviewer #4:** Lines 177 and 179, S1 table, S2 table: If the authors really followed the methods described by Miotto et al. (ref 8), as they claim, these supplementary tables should be deleted. Likewise, the text from line 174 to 179 (“briefly, used in amplification…” to “synthesized by Eurofins scientific (S2 table)” can be deleted.
   However, after reading the works of Miotto et al. (ref 8), I realize that Miotto et al. performed whole genome sequencing using Illumina, while the authors of the present manuscript performed nested PCR followed by Sanger sequencing. The authors should clarify what they mean by performing nested PCR “following a method previously described by Miotto et al. (8).”

**Response:** Thanks for the comment, we used nested PCR using primers developed following a study by Miotto *et al.,* 2015. This has been rectified in the revised manuscript.

5. **Comment Reviewer #4:** S1 table: The authors state that nested PCR was performed (lines 171-172). What does the hyphen in “second round PCR” for fd and arps10 genes mean? Apparently, the second round PCR was not performed for these two genes, as it can be seen in S2 table. Please revise and clarify the text.

**Response:** Second round PCR was not performed for *fd* and *arps10* gene, this has been rectified in the revised manuscript.

6. **Comment Reviewer #4:** S1 and S2 tables: Please add the source of the PCR protocol and primer sequences. Unlike what is indicated in the text (line 173), it does not seem to have been developed by Miotto et al. (ref 8), who used Illumina.

**Response:** The primers used in this study were developed following a study by Miotto *et al.,* 2015. The PCR conditions optimized for each of the genes (*fd, arps10, pfmdr-2, pfcrt*) prior to DNA amplification. This has been included in the revised manuscript.

7. **Comment Reviewer #4:** Discussion: The presence of artemisinin-resistant Pf parasites is not fully established in Africa, with the possible exception of northern Uganda in recent years, i.e. since 2017 (see Balkagala et al., N Engl J Med 2021;385:1163-1171), rendering any argument for a genetic background uncertain. The hypothesis is all the more difficult to test based on 142 isolates, of which 7 presented Pfk13 mutations. Moreover, the key Pfk13 mutations found by Balikagala et al., A675V and C469Y, were not reported by the authors of the present manuscript. These considerations should be discussed.

**Response:** The discussion section of the manuscript addresses the possible effect of introduction of artemisinin combination therapies on development and spread of artemisinin resistance. Our results do indicate that over time after the introduction of artemisinin combination therapies the number of K13 mutations whose role in artemisinin resistance is unknown increased. This finding is in line with many other studies done in sub-Saharan Africa (Ocan *et al.,* 2019; Ndiwa *et al.,* 2021). However, more recent studies have started reported K13 mutations, 675V, 469Y that are validated to cause artemisinin resistance in South east Asia however the role of this mutations in causing artemisinin resistance in *P. falciparum* parasites in sub-Saharan Africa still remains unknown.

These considerations have been incorporated in the revised manuscript.

8. **Comment Reviewer #4:** Line 73: artemisinin-based combination therapies (ACTs)

**Response:** This has been rectified in the revised manuscript to Artemisinin combination therapies (ACTs)

9. **Comment Reviewer #4:** Lines 74-75, “despite the threat of parasite resistance to both artemisinin and piperaquine components of the combination in SEA”: How about other drug partners of artemisinin derivatives: decreased susceptibility (or resistance) to mefloquine and lumefantrine and resistance to amodiaquine and sulfadoxine-pyrimethamine in SEA? This part of the sentence should be rewritten.

**Response:** In sub-Saharan Africa, artemisinin combination therapies (ACTs) remain highly efficacious in malaria treatment despite the threat of parasite resistance to both artemisinin (WHO malaria report, 2020; Thanh *et al.,* 2017) and other components of the combination in South east Asia (Leang *et al.,* 2013).

This has been corrected in the revised manuscript.

10. **Comment Reviewer #4:** Line 75: southeast Asia (SEA); The authors should decide whether they will use SEA throughout the text or not. See lines 83, 86, 91.

**Response:** This has been corrected in the revised manuscript. We have used southeast Asia throughout the revised manuscript.

11. **Comment Reviewer #4:** Lines 79-80: China and Bangladesh are not part of southeast Asia.

**Response:** The correction has been effected in the revised manuscript.

12. **Comment Reviewer #4:** Lines 80-83, “like previous antimalarial agents” and “…after first emerging in southeast Asia”: What are these “previous antimalarial agents?” Chloroquine resistance was first reported in South America (almost at the same time as in southeast Asia). The same is true with pyrimethamine. These sentences need to be revised.

**Response:** This has been corrected in the revised manuscript.

13. **Comment Reviewer #4:** Line 85: Unique parasite genetic background supportS independent emergence…

**Response:** This has been corrected in the revised manuscript.

14. **Comment Reviewer #4:** Line 90: P. falciparum (instead of “Pf”)

**Response:** This has been corrected in the revised manuscript

15. **Comment Reviewer #4:** Lines 87-92: The authors repeat the same idea in these two sentences (“Miotto et al. reported…” and “The background mutations…”). The examples (fd-D193Y, aps10-V127M, mdr2-T484I, crt-I356T, crt-N326S) can be incorporated in the first sentence; the second sentence should be omitted.

**Response:** This has been corrected in the revised manuscript

16. **Comment Reviewer #4:** Line 93: in sub-Saharan Africa (the abbreviation SSA was not introduced earlier in the text).

**Response:** This has been corrected in the revised manuscript

17. **Comment Reviewer #4:** Line 102, “prolong effective half-life”: The meaning of “half-life” in this sentence is not clear.

**Response:** This has been corrected in the revised manuscript

18. **Comment Reviewer #4:** Line 113: artemisinin-based combination therapies

**Response:** This has been corrected to read, artemisinin combination therapies

19. **Comment reviewer #4:** Line 128: ethylenediaminotetraacetic acid (EDTA)

**Response:** This has been corrected in the revised manuscript

20. **Comment Reviewer #4:** Line 130: It is not clear in what form blood samples were stored (frozen whole blood? Imbibed onto filter paper?).

**Response:** The blood samples were stored as frozen whole blood. This has been corrected in the revised manuscript

21. **Comment Reviewer #4:** Line 134: collected in 2005 and 2015 by Dorsey et al. (16) and Kamya et al. (17), respectively,…

**Response:** This has been corrected in the revised manuscript

22. **Comment Reviewer #4:** Line 136: P. falciparum

**Response:** This has been corrected in the revised manuscript

23. **Comment reviewer #4:** Line 138: histidine rich protein 2 (not in italics)

**Response:** This has been corrected in the revised manuscript

24. **Comment reviewer #4:** Line 139, “as described earlier”: Please provide a reference citation.

**Response:** This has been corrected in the revised manuscript

25. **Comment reviewer #4:** Lines 139-144, from “Briefly, 10 µl of whole blood…” to “a pink line does not appear (18)”: These sentences which describe in detail how to perform a RDT are not necessary. They should be deleted. Ref 18 can be placed at the end of the previous sentence after “as described earlier by following the manufacturer’s guidelines.”

**Response:** This has been corrected in the revised manuscript

26. **Comment Reviewer #4:** Line 153: Please add the manufacturer, city, country of Nano drop.

**Response:** Thermo Scientific, Wilmington, Delaware, USA. This has been corrected in the revised manuscript

27. **Comment reviewer #4:** Line 153 “samples with > 50 ng of Pf parasite DNA”: The authors did not describe how they separated parasite DNA from human DNA in their samples. They should either describe the procedure, cite a reference that explains the procedure, or revise the sentence (that they are referring to the mixture of parasite and human DNA). Same comment for line 162 (“50 ng/µL of Pf DNA”).

**Response:** Human DNA was not separated from parasite DNA therefore the DNA used was mixed human and parasite DNA.

28. **Comment Reviewer #4:** Lines 160-165: If the authors followed the PCR protocol described by Ariey et al., as they state in lines 158-159, lines 160-165 can be deleted.

**Response:** This has been corrected in the revised manuscript

29. **Comment Reviewer #4:** Lines 181-183. This sub-title can be deleted. There is only 1 short sentence under this sub-section.

**Response:** This has been corrected in the revised manuscript

30. **Comment Reviewer #4:** Line 184: What is “ambient controlled conditions”?

**Response:** This has been corrected in the revised manuscript

31. **Comment Reviewer #4:** Line 199: Ile492Val

**Response:** This has been corrected in the revised manuscript

32. **Comment Reviewer #4:**  Line 200: Were electropherograms deposited in the GenBank?

**Response:** We did not submit electropherograms in the GenBank. However, we have submitted the gene sequences to the GenBank

33. **Comment Reviewer #4:** Lines 207: The authors apparently eliminated samples with less than 50 ng DNA (see line 153). Of 200 samples, how many had more than 50 ng DNA? This information is required in the main text.

**Response:** All the 200 samples had 50 ng and more of the DNA.

34. **Comment Reviewer #4:** Lines 216-217, “Five K13 non-synonymous SNPs E643K, E596V, S600C, W565X, and A578S were detected in Pf parasite samples collected in 2015”: A very similar sentence is in lines 210-213. The sentence in lines 216-217 can be deleted to avoid repetition.

**Response:** This has been corrected in the revised manuscript

35. **Comment Reviewer #4:** The authors mention several times W565X, in the main text and in Table 3. Please specify that amino acid X refers to stop codon.

**Response:** Yes, amino acid X refers to a stop codon. We sent the sample K045 for re-analysis (sequencing) and new sequence out-put did not contain W565X, this mutation has therefore been removed from the revised manuscript.

36. **Comment Reviewer #4:** Lines 238: The authors eliminated samples with less than 50 ng DNA (see line 153). Of 200 samples, how many had more than 50 ng DNA? The success rate in percentage for each marker should be presented in parentheses after each number of successful sequencing.

**Response:** All the samples included in our study had 50 ng or more of the DNA. There is no need of indicating this for each gene

37. **Comment Reviewer #4:** Line 242: Ile492Val

**Response:** This has been corrected in the revised manuscript

38. **Comment Reviewer #4:** Line 250: resistance HAS

**Response:** This has been corrected in the revised manuscript

39. **Comment reviewer #4:** Line 253: Ile492Val

**Response:** This has been corrected in the revised manuscript

40. **Comment Reviewer #4:** Line 257: ACT or artemisinin-based combination therapies

**Response:** This has been corrected in the revised manuscript

41. **Comment Reviewer #4:** The references should be in the same format. The first letters of the article title are in capital letter in Ref 2, 7, 12, 13 and others.

**Response:** This has been corrected in the revised manuscript

42. **Comment Reviewer #4:** Ref 20 journal: Nature

**Response:** This has been corrected in the revised manuscript

43. **Comment Reviewer #5:** The revised manuscript and the tacked changed revised manuscript are two different manuscripts. Which makes it extremely difficult to judge the manuscript. Authors are strictly advised to follow the journal rules and submit the uniform manuscript.
    Line 56 -61 of the track change version was entirely absent in the revised manuscript.

**Response:** This has been corrected in the revised manuscript

44. **Comment Reviewer #5:** The proportion of Kelch13 mutant alleles in 2005 and 2015 were not clearly understood in the abstract section. It will be great if the authors present it in percentage value.

**Response:** This has been corrected in the revised manuscript

45. **Comment Reviewer #5:** Line 79-80; In 2018- 2019 partial artemisinin resistance was reported from India. Reduced susceptibility to artemisinin was also reported in 2021 from India. India is not a part of South-east Asian Countries. Mentioned it in the text and modify the write-up accordingly in line 81-84.

**Response:** This has been corrected in the revised manuscript

46. **Comment Reviewer #5:** Line 93; Use the full form of SSA when you use it for the first time in your manuscript.

**Response:** This has been corrected in the revised manuscript

47. **Comment Reviewer #5:** Do the authors check the clonality of the infection? For confirmation of P. falciparum, authors have used the RDTs and Microscopy. How do they check the multiplicity of infection which is supposed to be a very important parameter as per their proposed objectives.

**Response:** Yes, from the analysis of peaks of the sequence data. Multiplicity of infection was assessed using the peak heights, where a mixed genotype was confirmed if the minor peak was higher than a third of the major peak.

48. **Comment Reviewer #5:** Line 134-135; Apart from “randomization” do the authors have considered any specific inclusion and exclusion criteria for sampling.

**Response:** In addition, to randomization we only included samples with parasite DNA ≥ 50ng/ml

49. **Comment Reviewer #5:** Line 172; fcrt should be corrected as pfcrt.

**Response:** This has been corrected in the revised manuscript

50. **Comment Reviewer #5:** Depending on the findings of 2005 and 2015 SNP data, authors are requested to find some suitable statistical validation of their results.

**Response:** This was a descriptive study, reporting on the various mutations which were detected in the parasite samples collected in 2005 and 2015

51. **Comment Reviewer #5:** The revised discussion section was much improved.

**Response:** Thanks, much appreciated.
