# Supplementary material for: Prevalence of arps10, fd, pfmdr-2, pfcrt and pfkelch13 gene mutations in Plasmodium falciparum parasite population in Uganda
Source: PLoS One. 2022 May 5;17(5):e0268095. doi: 10.1371/journal.pone.0268095 (PMC9070901; doi:10.1371/journal.pone.0268095)
Supplement: Attachment — Submitted filename: RESPONSE TO THE REVIEWER_dec21.doc [file pone.0268095.s004.doc]

**RESPONSE TO THE REVIEWER’S COMMENTS**

**Reviewer #1: Comments:**
The MS “Prevalence of arps10, fd, mdr-2, pfcrt and pfkelch13 gene mutations in Plasmodium falciparum parasite population in Uganda” by Moses Ocan et al., presents useful molecular surveillance information on the polymorphisms in the genes responsible for driving development of resistance to artemisinins.

**Response:** Thanks for the comment

**Reviewer #1: Comments:** Major comments:
1) Abstract/Results

It is difficult to follow the results description in the Abstract and Results as there is no clear demarcation between the SNP prevalence before 2005 and after 2015, because the authors report the prevalences in the pooled sample set. In my view, the analysis (in the Results and the Abstract) should be presented for each cohort separately (as a Table) and basic statistical analysis (presenting P values and confidence intervals) should be done to claim any significant difference in the point prevalences of SNPs before and after introduction of the ACTs. In the current version it is not clear if the following statement in lines 52-53 can be made “This is an indicator of the role Artemisinin combination therapies play in altering P. falciparum parasite genotype and potentially driving resistance development”. I suggest to change it emphasizing the importance of molecular surveillance in order to detect emerging resistance.

**Response:** Re-analysis of the data has been done to reflect prevalence of mutations in the different years. For K13 SNPs overall prevalence is 4.2% (6/142) while in 2005 (1.4%, 2/142) and 2013 (2.8%, 4/142). For the *pfmdr-2* mutations, overall prevalence is 39.8% (74/186) while in 2005 (2.2%, 4/186) and in 2013 (37.6%, 70/186). For *arps10* mutations, overall prevalence is 2.7% (2/74) while in 2013 (2.7%, 2/74) and this mutation was not detected among samples collected in 2005. There was no significant relationship in the prevalence of mutations in the K13 (p=0.087) and *arps10* (p=0.146) genes for samples collected in 2005 and 2013. There was a significant relationship in the prevalence of mutations in the *pfmdr-2* gene, p<0.001 among samples collected in 2005 and 2013.

Detailed results on this analysis is presented in Table 4 in the revised manuscript.

This revision has been effected in the revised manuscript.

**Reviewer #1: Comments:** 2) Introduction still needs more information on ACTs used in these areas, their efficacies and any evidence of resistance developing. Recommend to add references such as Assefa, D.G., Zeleke, E.D., Bekele, D. et al. Efficacy and safety of dihydroartemisinin–piperaquine versus artemether–lumefantrine for treatment of uncomplicated Plasmodium falciparum malaria in Ugandan children: a systematic review and meta-analysis of randomized control trials. Malar J 20, 174 (2021).

**Response:** Thanks for the comment, the requested information is mentioned in the first paragraph of the manuscript. However, we have added further information on the different ACTs used in Uganda. In Uganda, the recommended first-line and second-line agents in treatment of uncomplicated malaria are Artemether-lumefantrine (AL) and Dihydroartemisin-Piperaquine (DHP-PPQ) respectively (MoH, 2011). A recent systematic review reported a slightly better efficacy of DHP-PPQ compared to AL in the treatment of uncomplicated malaria among children under five years in Uganda (Assefa *et al.,* 2021).

This has been incorporated in the revised manuscript.

**Reviewer #1: Comments:** 3) Information on molecular markers of artemisinin resistance in Africa needs to be added, for example, found in recent refs: Tumwebaze PK, Katairo T, Okitwi M, Byaruhanga O, Orena S, Asua V, Duvalsaint M, Legac J, Chelebieva S, Ceja FG, Rasmussen SA, Conrad MD, Nsobya SL, Aydemir O, Bailey JA, Bayles BR, Rosenthal PJ, Cooper RA. Drug susceptibility of Plasmodium falciparum in eastern Uganda: a longitudinal phenotypic and genotypic study. Lancet Microbe. 2021 Sep;2(9):e441-e449.
4) There is also a similar study (Conrad MD, Nsobya SL, Rosenthal PJ. The Diversity of the Plasmodium falciparum K13 Propeller Domain Did Not Increase after Implementation of Artemisinin-Based Combination Therapy in Uganda. Antimicrob Agents Chemother. 2019;63(10):e01234-19.) conducted in the same areas, where the authors found “No differences in diversity following implementation of ACT use were found at any of the seven sites, nor was there evidence of selective pressures acting on the locus. Our results suggest that selection by ACTs is not impacting on K13PD diversity in Uganda”. This ref and information should be discussed in Introduction and Discussion.

**Response:** According to a number of studies, K13 mutations in Africa are very diverse and its probable that each study is likely to find slightly different K13 mutations. A statement on the diversity of K13 mutations in sub-Saharan Africa is provided in the earlier version of the manuscript and we think that this is sufficient information based on the current evidence around K13 mutations in Africa. Additionally, a paper by Conrad *et al.,* 2019, reports no increase in the diversity of K13 mutations however, like our current study the article finds a general increase in the prevalence of K13 mutations in the period after introduction of ACTs in malaria treatment in Uganda. A statement on the relationship between introduction of ACTs and the diversity of K13 mutations has been incorporated in the revised manuscript.

This information has been incorporated in the revised manuscript

**Reviewer #1: Comments** 5) Materials and Methods. Study collection times need to be clarified.
Lines 124-125: Suggest specify enrolment time as in ref 16: “… with enrolment conducted from November 2004 to April 2005 in Kampala (16).

**Response:** Yes, sorry for the lack of clarity on this. We picked samples for our current study from those which were collected in 2005. This statement has been incorporated in the revised manuscript.

**Reviewer #1: Comments:** Ref 17 has incorrect publication year 2020 instead of 2015 in
Kamya MR, Arinaitwe E, Wanzira H, Katureebe A, Barusya C, Kigozi SP, et al. Malaria Transmission, Infection, and Disease at Three Sites with Varied Transmission Intensity in Uganda: Implications for Malaria Control. Am J Trop Med Hyg. 2020;92(5):903-12.
It should be “Malaria transmission, infection, and disease at three sites with varied transmission intensity in Uganda: implications for malaria control.

**Response:** Thanks for the comment. This has been corrected in the revised manuscript.

**Reviewer #1: Comments:** Kamya MR, Arinaitwe E, Wanzira H, Katureebe A, Barusya C, Kigozi SP, Kilama M, Tatem AJ, Rosenthal PJ, Drakeley C, Lindsay SW, Staedke SG, Smith DL, Greenhouse B, Dorsey G. Am J Trop Med Hyg. 2015 May;92(5):903-12”.
This study collection time is specified in this reference as “between August 2011 to September 2013” not “to 2018” as stated in the MS. This requires further clarification.

**Response:** Thanks for the comment. We are sorry for the lack of clarity on this. The dates for sample collections have been corrected. For our study we analyzed samples collected in 2013. This has been clarified in the revised manuscript.

**Reviewer #1: Comments:** As only a subset from these studies being used in the present study the detailed description of how many samples from each site were included in either cohort would be beneficial.

**Response:** In our current study only samples from Jinja and Kampala were used. Of which 100 samples each from Jinja and Kampala were picked.

This has been provided in the revised manuscript.

**Reviewer #1: Comments:** 6) Line 134 “For our study, 100 P. falciparum infected blood samples each collected in 2005 and 2015 by Dorsey et al., (16) and Kamya et al., (17), respectively were randomly selected for inclusion. In addition, only samples with ≥50ng/µl of DNA were included in the study.”
The statement needs to be rephrased as samples were not exactly collected in 2005 or 2015. This needs to be addressed throughout the entire text.

**Response:** Thanks for the comment, a study by Dorsey *et al.,* (16), a single-blind randomized clinical trial was conducted between November 2004 and June 2006. In our current study, were accessed 100 blood samples collected in 2005. For a study by Kamya *et al.,* (17), the study was conducted from August 5, 2011 to September 30, 2013. We accessed 100 blood samples collected in 2013. This has been corrected in the revised manuscript.

**Reviewer #1: Comments:** How many samples from each site were included and how randomization was achieved?
Also suggest to change to “100 randomly selected samples with ≥50ng/µl of DNA were included into the study” .

**Response:** A study by Kamya *et al.,* (17) was part of a larger PRISM 2 study which was conducted in three districts of Jinja, Kanungu and Tororo while the study by Dorsey *et al.,* (16) was conducted only in Kampala. For our current study, only 100 samples each from Jinja and Kampala were used. This has been incorporated in the revised manuscript.

**Reviewer #1: Comments:** 6). Line 155: It is not clear if the primers for K13 amplification were designed by the authors or just synthesized based on previously published ones by Ariey et al. 2014? Did authors designed the primers for other genes SNPs?

**Response:** Thanks for the comment. For K13 gene, we synthesized the primers based on previously published primers in a study by Ariey et al., 2014. For *pfmdr-2, arps10, fd* and *pfcrt* genes, the primers published in a study by Miotto *et al.,* were double checked using NCBI primer 3 software. The primers reported in a study by Miotto *et al.,* were then synthesized. All the primers were synthesized by Eurofins scientific. This has been corrected in the revised manuscript.

**Reviewer #1: Comments** :7) Supplement A includes the tables also included in S1 and S2 supplementary files, which is redundant.

**Response:** Thanks for the observation, ‘Supplement A’ has been removed/deleted from the manuscript files in the revised submission.

**Reviewer #1: Comments:** 8) Lines 193 and 218” Authors refer to carrying “amplicon sequencing”, perhaps, meaning “PCR amplicon”, which is a little bit confusing since this term is typically used for deep amplicon sequencing, where the libraries are generated from a PCR product and sequenced to identify rare mutations. In this study it appears that authors have carried out Sanger sequencing of PCR products. If that is the case, I suggest, that the authors remove “amplicon” and refer to their sequencing methodology as “Sanger sequencing of PCR products”.

**Response:** Thanks for the comments, this has been corrected in the revised manuscript. The statement now reads, ‘Sanger sequencing of PCR products’.

**Reviewer #1: Comments:** It also follows, that it is not possible to determine the multiplicity of infections by analysing the Sanger sequencing chromatograms peaks (e.g., of K13 genes). It would be possible to determine the dominant alleles, but this method lacks sensitivity and unable to determine minor alleles (as outlined in Zhong, D., Koepfli, C., Cui, L. et al. Molecular approaches to determine the multiplicity of Plasmodium infections. Malar J 17, 172 (2018). Analysis of highly polymorphic markers (genes or microsattelites) is used to estimate MOI.
9) Lines 302… The statement in conclusions is correct but without statistical analysis can be misleading: “The proportions of pfkelch13, arsp10, and mdr-2 gene mutations were higher in P. falciparum parasites collected a decade after introduction of artemisinin combination therapies in malaria treatment in Uganda.”

**Response:** The limitation of analyzing Sanger sequencing chromatogram peaks in determining multiplicity of infections has been included in the revised manuscript. We have performed statistical analysis of the data to support the statement in the conclusion.

Minor comments:
**Reviewer #1: Comments:** 1) For consistency, the genes’ abbreviations need to be the same throughout the text, for an example mdr-2 or pfmdr2. There are typos in arps10 gene name in lines 85 and 109.

**Response:** Thanks for the comment, this has been adjusted in the revised manuscript.

**Reviewer #1: Comments:** 2) Likewise, aminoacid abbreviations used in some cases are 3-letter ones whereas in others are 1-letter abbreviation -needs to be consistent throughout the text.

**Response:** This has been corrected in the revised manuscript.

**Reviewer #1: Comments:** 2). It is more appropriate to use single nucleotide polymorphism (SNP) term rather than mutations, for an example when referring to K13 polymorphisms.

**Response:** This has been adjusted in the revised manuscript

**Reviewer #1: Comments:** 3). Ref 13 and 27 are the same.

**Response:** Thanks for the comment, this has been corrected in the revised manuscript.

**Reviewer #1: Comments:** 4) Lines 96 “Wide spread chloroquine resistance followed by its subsequent withdrawal…” suggest to change to “Wide spread of resistance to chloroquine followed by its subsequent withdrawal…”

**Response:** Thanks, this has been changed to “Wide spread of resistance to chloroquine followed by its subsequent withdrawal…”, in the revised manuscript.

**Reviewer #1: Comments:** Line 148: add GmbH and the country information to Qiagen company reference in the following sentence: “P. falciparum genomic DNA was extracted using QIAamp DNA mini kit (Qiagen, GmbH, Germany).

**Response:** Thanks for the comment, this has been revised to “P. falciparum genomic DNA was extracted using QIAamp DNA mini kit (Qiagen, GmbH, Germany) in the corrected manuscript.

**Reviewer #2: Reviewer’s comments:**
The authors describe the prevalence of Pfkelch 13 gene mutations and other background genetic mutations thought to associate with artemisinin resistance. The author utilised available samples at their disposal to answer the research question. While I appreciate the efforts of the authors, I shall request that the following corrections be made before acceptance:
#1- The author should state clearly if the mutations found in the Pfkelch 13 gene were among associated to artemisinin resistance. This should be clear as from abstract through discussion.

**Response:** Thanks for the comments, in our manuscript this information is clearly stated in the discussion section. The K13 mutations found in our study have not among those that have been validated to be associated with artemisinin resistance.

**Reviewer #2: Reviewer’s comments:** #2- The author should so kindly introduce some statistical analysis like fishers’ exact test or chi square to compare the proportion of mutations before and after introduction of ACT in Uganda in his samples. This is important!!!

**Response:** Thanks, we have performed statistical analysis and have incorporated the findings in the results section of the manuscript.

**Reviewer #2: Reviewer’s comments** Line 42#- the sentence should be corrected.

**Response:** Thanks, we have corrected the sentence.

**Reviewer #2: Reviewer’s comments**: Line 81-82# I went through the reference provided by the author and it appears that it didn’t support the statement. The author should find an appropriate reference to that statement.

**Response:** Thanks for the comment, we have provided an alternative reference in the revised manuscript.

**Reviewer #2: Reviewer’s comments**: Line 217#. Could the author please make a clear table showing the mutations that occurred in 2005 and that which occurred in 2015. The table is not interesting in its current form and does not represent the line of thoughts in the paper.

**Response:** Thanks for the comment, the mutations that occurred in 2005 and 2015 have been clearly indicated in the table in the revised manuscript.

**Reviewer #2: Reviewer’s comments**: Line 254 -225#- The author seems to contradict his basis for accessing the background mutations. In line 81-86, the author mentioned that the background mutations were associated with artemisinin resistance in different geographical areas in Southeast Asia, but here he presents that their role in artemisinin resistance is not known. I suggest that the authors should think around this and rephrase accordingly.

**Response:** Thanks for this observation however, this statements are not contradictory. A study by Miotto *et al.,* 2011 confirmed the role of certain background mutations; *fd-*D193Y (ferredoxin gene), *arps10-*V127M (apicoplast ribosomal protein S10 gene), *pfmdr2-*T484I (multidrug resistance protein 2 gene), *crt-*I356T (chloroquine resistance transporter gene) and *crt-*N326S (chloroquine resistance transporter gene) in supporting the development of K13 mutations associated with artemisinin resistance. In our study we found only one mutation *arsp10-*V127M in one parasite sample collected in 2013 that had been previously reported in a study by Miotto *et al.* However, different background mutations from those reported by Miotto *et al.,* were found in other genes and this are the mutations which the statement is referring to. This is because the role of the different background mutations that we found in our study is not known as they have not been confirmed/validated. This has been clarified in the revised manuscript.

**Reviewer #2: Reviewer’s comments:** Line 298-300# - The author should support the statement with statistics.

**Response:** Thanks, we have performed statistical analysis and provided the results to support the statement.

**Reviewer #2: Reviewer’s comments:** Line 295-300#- The author should fit in one or two implications of his findings in the conclusion to make a bit stronger.

**Response:** Thanks, this has been effected in the revised manuscript.
