# Supplementary material for: Prevalence of arps10, fd, pfmdr-2, pfcrt and pfkelch13 gene mutations in Plasmodium falciparum parasite population in Uganda
Source: PLoS One. 2022 May 5;17(5):e0268095. doi: 10.1371/journal.pone.0268095 (PMC9070901; doi:10.1371/journal.pone.0268095)
Supplement: Attachment — Submitted filename: RESPONSE TO REVIEWER.doc [file pone.0268095.s006.doc]

**RESPONSE TO REVIEWER’S COMMENTS ON MANUSCRIPT PONE-D-21-33254R1**

We thank the reviewers for the comments on our manuscript, addressing this comments have improved the manuscript. Here below is the summary of our responses to the comments raised by the reviewers.

**Comment Reviewer #1:** The manuscript has been substantially improved since last revision. I believe the data presented in the paper are important and worthy of publication, but the paper still requires some work.
Below are some comments, which I believe still need to be addressed.
Major comments
(Lines numbers are based on numbers in the pdf version containing track changes.)

**Response:** Thanks for the observation, this has been addressed in the revised manuscript

**Comment Reviewer #1:** 1). Authors have now added Table 4 to the Results section that shows the breakdown in prevalence of mutations in years 2005 and 2013, which is appreciated. However, there are errors in calculations for the 2005 and 2013 prevalence for all molecular markers. In this table the overall frequency of SNPs (for both years 2005 and 2013) is calculated correctly as total number of SNPs divided by total number of samples analysed for both years. However, when the authors calculate the prevalence in years 2005 or 2013, separately, the authors also use the total number of samples in the denominator, rather than using the number of samples from the respective year. This is not correct.
For an example, for SNP prevalence in K13 gene the total number of samples in which K13 gene was successfully sequenced was 142, of which 51 samples were collected in 2005 and 91 were collected in 2013. For overall (2005 and 2013) prevalence of K13 SNPs authors use the following calculation:
[6 SNPs)/142]*100%=4.2%, which is correct. However, for the year 2005 the prevalence is calculated as (2/142)* 100%=1.4% and for 2013 as (4/142)* 100%=2.8%, which is not correct. It should be calculated as (2/51)*100%= 3.9% and (4/91)*100%=4.4% for 2013, respectively. The same considerations and corrections would apply to other molecular markers analysed in the study (pfmdr-2 and arps10). Consequently, the contingency analysis for each SNP for 2005 and 2013 should be done with P-values calculated using exact Fisher’s test with 95% confidence intervals also recalculated for all markers/SNPs. This would also may change the conclusions reached in the paper and mentioned in the abstract.
I also suggest to include the number of samples analysed for every molecular marker gene for each year in the table 4 for ease of understanding as shown below:
Characteristic Molecular Marker/SNPs Collection Year Prevalence, % (n/N) P-value 95% CI
Pfkelch13 Overall 4.2 (6/142)
2005 1.4 3.9 (2/51)
2013 2.8 4.3 (4/91)

**Response:** Thanks for the comments, the re-analysis has been done using Fisher’s Exact test and the results are presented in Table 4. We have included the requested numbers in the revised table.

**Comment Reviewer #1:**  2). Lines: 37 (abstract), 128, 232, 236, 241, 280, 283, 285, 294. In these lines there are still references to 2015, which is incorrect as authors have confirmed that it should be 2013, the year when samples were collected. Could authors please check this for consistency throughout the text.

**Response:** Thanks for the observation. We have corrected the year to 2013 all through the revised manuscript.

**Comment Reviewer #1:** 3). Lines 233-239: When describing the SNPs in Pfkelch13 gene, authors should refer to Table 3 initially, and then to Table 4 when describing numbers and prevalence in years 2005 and 2013. I also could not find Tables 1 and 2 in the main Text. Could authors please check and change the Table numbers if this is the case.

**Response:** Tables 1 and 2 are referenced in the main text under the methods section but provided as additional material. Revision has been made on reference to Table 3 first when describing the Pfkelch13 gene.

**Comment Reviewer #1:** 4). Authors might want to consider changing the titles for the Table 3 “Single nucleotide polymorphisms identified in pfkelch13 gene in samples collected in Kampala and Jinja, Uganda in 2005 and 2013.” Also, in footnotes in Table 3 it would be good to add the year when polymorphism was observed in those samples.
Also, a suggestion for Table 4 title would be “Prevalence of single nucleotide polymorphisms in pfkelch13, Pfmdr-2 and Pfarps 10 genes in Plasmodium falciparum samples collected in Kampala and Jinja, Uganda in 2005 and 2013”, instead of “Table 4: Distribution of the P. falciparum parasite mutations by year of sample collection, 2005 and 2013 (K13, n=142; pfmdr-2, n =186; arps10, n = 74)”.
I suggest to remove the word “different” from description of SNPs in pfmdr-2 and arps 10 genes, because it includes the SNPs at 484 and 127 in those genes reported by Miotto et al., 2015. The explanation in the text would be sufficient.

**Response:** This correction has been effected in the revised manuscript

**Comment Reviewer #1:**  5) Abstract:
“In Uganda, Artemether-Lumefantrine and Artesunate are recommended for uncomplicated and 31 severe malaria respectively, but are currently threatened by parasite resistance”-I think the introductory sentence should be reworded as at present the data do not support resistance threat for this combination in Africa.
Also, suggest to replace “transcriptional” with “epigenetic” factors, implying changes in transcription.
In the abstract the actual SNPs, which were analysed in the present study¬ in the molecular markers genes should be stated.
The sentence “Overall, prevalence of SNPs in pfmdr-2 gene was 39.8% (74/186), of this 2.2% (4/186), 37.6% (70/186) occurred in 2005 and 2013 samples respectively” is misleading as it only refers to 2 “other” SNPs Y423F and I492V (not mentioned as Southeast Asian background mutations), but not to the SNP at pfmdr-2 T424I SNP reported by (Miotto et al., 2015). This need to be clarified in the abstract.
The prevalence numbers for all SNPs need to be recalculated with the number of samples analysed in the respective year used in the denominator. These may change the conclusion “There were more pfkelch13, arps10 and pfmdr-2 SNPs gene mutations in P. falciparum parasites collected a decade (2015) after introduction of Artemisinin combination therapies in malaria treatment.”

**Response:** The introductory sentence is appropriate and current data does support resistance threat to artemisinin agents in Africa as shown in a study by Balikagala *et al.,* 2021. This sentence has been maintained in the revised manuscript.

The word ‘transcriptional’ has been replaced with ‘epigenetic’ in the revised manuscript.

The sentence “Overall, prevalence of SNPs in pfmdr-2 gene was 39.8% (74/186), of this 2.2% (4/186), 37.6% (70/186) occurred in 2005 and 2013 samples respectively is appropriate. However, we have removed the word ‘overall’ in the revised manuscript.

The prevalence of all the SNPs has been recalculated however, the direction of the findings remained the same and thus the conclusion “There were more pfkelch13, arps10 and pfmdr-2 SNPs gene mutations in P. falciparum parasites collected a decade (2015) after introduction of Artemisinin combination therapies in malaria treatment.” Has been maintained in the revised manuscript.

**Comment Reviewer #1:** 6). I believe that Discussion needs to be amended to reflect the following points:
1) The majority of samples before and after introduction of the ACTs are the wild type with only few SNPs identified in a very low fraction of samples (needs to be recalculated and presented). Importantly, they were different SNPs detected after introduction of ACTs, compared to those detected prior to, albeit all occurred at very low frequencies before and after introduction of the ACT.
2) As the K13 SNPs detected before and after introduction of ACTs are different it has to be stressed when making a comparison of K13 SNPs and conclusion that we see “There were more pfkelch13, arps10 and pfmdr-2 SNPs gene mutations in P. falciparum parasites collected a decade (2015) after introduction of Artemisinin combination therapies in 64 malaria treatment”. (lines 62-64) and in the discussion (Lines 320-322): “Additionally, our study found a general increase in the prevalence of K13 SNPs in samples collected after (2013) introduction of ACTs in malaria treatment in Uganda. It is likely that statistical analysis of the data would support this conclusion.
3) Importantly, there were no SNPs that were previously reported in Uganda “Balikagala et al., (34) demonstrated delayed artemisinin clearance among parasites carrying 675V and 469Y mutations in Uganda” (line 328-329) found in this study. These findings and their implications for the efficacy of the ACTs and potential for the resistance should be discussed further.
4) Absence of mutations in pfmdr-2 (T484I) and arps10 (V127M) in Africa was previously noted by Miotto et al 2015, with conclusion that these background mutations are specific to Southeast Asia. These findings have been subsequently confirmed by other recent studies that can be added to the discussion (Diakité, Seidina A S et al. “A comprehensive analysis of drug resistance molecular markers and Plasmodium falciparum genetic diversity in two malaria endemic sites in Mali.” Malaria journal vol. 18,1 361. 12 Nov. 2019, doi:10.1186/s12936-019-2986-5). Also findings on pfmdr-2 SNPs from Hussien M at al., Antimalarial drug resistance molecular makers of Plasmodium falciparum isolates from Sudan during 2015-2017. PLoS One. 2020 Aug 20;15(8)) should be discussed.
5) There were no mutations in Pfcrt N326S observed in the present study, whereas in Hussian et al there were 46.3% 96/207 samples carrying this SNP. This needs to be discussed.
6) Discussion on the importance in rise of other mutations in pfmdr-2 I492V 18.8? (35/?) 12.0 -39.0 Y423F 18.8 ?(35/?) needs to be added as these SNPs are the only ones that significantly more prevalent in 2013 (needs to be supported by stat. analysis).

**Response:** Thanks for the comment, the discussion has been amended to reflect the suggestions. The role the different background mutations identified from the genes need to be validated among the African *Plasmodium falciparum* parasites.

Minor comments
**Comment Reviewer #1:** Line 192: abbreviation PCR can be used as it has been previously defined. The same can be said about using SNP abbreviation throughout the text after it was first defined.

**Response:** Thanks, this has been corrected in the revised manuscript

**Comment Reviewer #1:** Lines 198: “double checked based on the 3D7 genome…” replace were tested

**Response:** Thanks, this has been revised in the corrected manuscript

**Comment Reviewer #1:** Line 234: (4.2%, 6/142) K13 single nucleotide polymorphisms (SNPs) were detected (Table 4). Of these, five (5) were non-synonymous and one (1) was a synonymous K13 mutation, (I587I).

**Response:** This has been corrected in the revised manuscript.

**Comment Reviewer #1:** Lines 235-236: four (54) (error), one (1) K13 non-synonymous mutations in the P. falciparum parasite samples collected 236 in 2015 and 2005 respectively.
In some sentences throughout the text numbers are duplicated in brackets in others are not. Please use consistent convention in the paper. Also, when listing SNPs it would be better to use their aa position numbers in the ascending order. For an example,

**Response:** Thanks for the observation, this has been corrected in the revised manuscript.

**Comment Reviewer #1:** Line 238-241: One (1) K13 non-synonymous SNP Y588N occurred in a P. falciparum parasite sample collected in 2005. Five (5) K13 non-synonymous SNPs A578S, E596V, S600C and E643K and were detected in P. falciparum parasite samples collected in 2015.
Also, please use the same order (ascending) in the Table 4.

**Response:** Thanks for the comment, this has been corrected in the revised manuscript.

**Comment Reviewer #1:** Line 276-278: Polymorphisms in the fd, pfmdr -2 and pfcrt genes that are markers of a genetic background where pfkelch13 mutations associated with slow artemisinin parasite clearance are likely to arise previously reported in Southeast Asia (Miotto, 20160 were not found in our study

**Response:** Thanks for the observation, the citation, Miotto et al., has been included into the sentence in the revised manuscript.

**Comment Reviewer #1:** Line 277: Pf mdr – 2 replace with pfmdr-2. Also, in some instances authors use pfkelch 13 and others K13 gene name. Please, use the same name throughout.

**Response:** Thanks for the comment, this has been corrected in the revised manuscript.
