# Supplementary material for: Prevalence of arps10, fd, pfmdr-2, pfcrt and pfkelch13 gene mutations in Plasmodium falciparum parasite population in Uganda
Source: PLoS One. 2022 May 5;17(5):e0268095. doi: 10.1371/journal.pone.0268095 (PMC9070901; doi:10.1371/journal.pone.0268095)
Supplement: Attachment — Submitted filename: RESPONSE TO REVIEWER.doc [file pone.0268095.s007.doc]

**RESPONSE TO REVIEWER’S COMMENTS ON MANUSCRIPT PONE-D-21-33254R2**

We are grateful to the reviewers for the comments raised on our manuscript. The comments have been addressed and here below is a summary of the point-by-point response to the different questions.

**Comment, Reviewer #1:** The authors have partially addressed some of my previously mentioned points. However, I believe that the paper still requires further improvements and authors should address the following points raised in my previous comments (please see below).
1). The Abstract/Results/Discussion (as previously mentioned)
The aim of the paper was stated in the Introduction (lines 113-116) as “We therefore, intended in this study to assess and 114 compare the prevalence of fd-D193Y, aps10-V127M, pfmdr2-T484I, crt-I356T, crt-N326S and 115 K13 mutations in P. falciparum parasites before (2005) and a decade (2013) after introduction of 116 artemisinin-based combination therapies for malaria treatment in Uganda (18)”. Therefore, the SNPs prevalences in those years should be calculated correctly as previously mentioned.

**Response:**

Thanks for this observation, actually the calculations were made as previously requested except that we forgot to remove the denominator ‘142’ and place the correct denominator used in the analysis, 51 (for 2005) and 91 (for 2013) for K13 mutations. This error has been corrected in the revised manuscript.

**Comment:** In the present revision authors have provided the break-down in numbers of samples analyzed in 2005 and 2013 and calculated the respective prevalence for each collection time, which are now shown in Table 4. However, the authors have not amended or changed the data in the Abstract and in the Results sections for the prevalence values for 2005 or 2013 to reflect that, and still used the total number of samples analysed for both years overall in the denominator.

**Response:**

Thanks for this observation, this was an editorial error as we forgot to adjust the denominators in the revised manuscript despite the fact that the analysis were done following the adjusted denominators as previously guided. We have adjusted the analysis as requested in the revised manuscript.

**Comment:** As the primary aim of this paper is to compare the prevalence of the SNPs in samples collected in 2005 and in 2013 (after introduction of ACTs), the respective prevalence should be calculated correctly. When reporting (throughout the paper), the prevalence values should be accompanied by the fraction (n/N) showing the number of samples with SNPs (n) and N number of samples analysed in this time period, as well as 95% confidence interval (CI). Also, when comparing these values, the authors should state if there is (or not) a statistically significant difference between the prevalence values in 2005 and 2013 (with P-value), which are calculated based on these numbers.

**Response:**

Thanks for the comment, this analysis are clearly stated in table 4. We have further provided the descriptive summaries for the different mutations in the results section of the revised manuscript.

**Comment:** Also, if authors could use the prevalence values in the Discussion rather than just mentioning a particular detected SNP, so the readers could put it in a context and appreciate the significance of the findings.

**Response:**

Thanks for the comment, this has been incorporated in the revised manuscript.

**Comment:** Please see below the specific examples (line numbers are as in the “Clean” version).

**Response:** This is noted

**Comment:** Lines 47-55 (Abstract) and throughout the Results section authors should amend the abstract and the result with the SNP prevalence data for 2005 and 2013 years respectively, where the number of SNPs is divided by the number of samples analysed in that particular time period (i.e. either in 2005 or in 2013), not the combined overall number of samples to match the “Prevalence” data shown in Table 4.

**Response:** Thanks, this has been effected in the revised manuscript

**Comment:** 2). I suggest Tables 1 and 2 should be renamed S1 and S2 as they are in the Supplementary material not in the main text and renumber Tables 3 and 4.

**Response:** The information in the tables S1 and S2 in the supplementary material is different from the information in tables 1 and 2 in the main text. We therefore, think the current numbering is appropriate and has been maintained in the revised manuscript.

3). Table 4. Please use consistent gene names nomenclature in the Table title and in the Table (i.e. Pfkelch 13 or K13).

**Response:** This has been noted and corrected in the revised manuscript

**Comment:** Column 2 and 3 have (%) in the columns’ headings but don’t actually show these percentages. These columns can be combined with the column “Prevalence, % (n)” if the number of samples (N) analysed per actual time period can be added “Prevalence, % (n/N)” as previously suggested.

**Response:** This has been noted however, from your previous comment you requested for a clear separation of number of samples which had and those which did not have mutations and we effected. We find it confusing to now request for merging the numbers again. We have however, taken note of the comment and made adjustments in table 4 as indicated in the revised manuscript.

4). Results section.
**Comment:** Lines 269 The sentence about the SNPs in pfmdr2 (“There was a significant relationship in the prevalence of mutations in the pfmdr-2 gene….”) should be included in the next section, as this section is about SNPs in K13 gene. Also, could authors state if there is a statistically significant difference between the prevalence values instead of "significant relationship")

**Response;**

This is noted and has been effected in the revised manuscript

**Comments:** Throughout the Results section the actual prevalence values from Table 4 should be used with confidence intervals and P-values for the prevalence values for 2005 and 2013, respectively. It is very important as it allows readers to appreciate the statistical analysis of the data. Also, if authors also could list codons in the ascending order when mentioning SNPs throughout the text (for example in line 278).

**Response:** Thanks for the comment, this has been effected in the revised manuscript

Discussion

**Comment:** If authors could use the prevalence values and sample numbers throughout the Discussion as well when commenting on identified SNPs and also compare with those in the referenced published literature.

**Response:** Thanks however, we this as a repetition of the results which are clearly stated in the results section. We have however, effected the adjustment as requested.

**Comment:** As the aim of the paper was to detect if the background mutations identified by Miotto et [al.in](http://al.in/) SEA are present in Uganda, it would be more logical to start the Discussion with the findings related to this primary aim and then continue discussing the secondary (also important) findings. I would suggest to rearrange the paragraphs in the Discussion and start the discussion with the text shown in lines 309-319.
319: “Plasmodium falciparum artemisinin resistance is a heritable trait with a genetic basis (7). Genome modification studies have shown that the impact of various K13 pfkelch13 mutations on P. falciparum artemisinin clearance and survival rates of ring stage parasites is dependent on the genetic background (27). The risk of emergence of K13pfkelch13 mutations associated with delayed artemisinin parasite clearance is thus driven by specific parasite genetic background (8, 314 28). In our current study polymorphisms in the fd, pfmdr-2 and pfcrt genes which support the rise of K13 mutations associated with delayed artemisinin clearance of P. falciparum parasites reported in Southeast Asia (8) were not found. However, SNPs in the pfmdr-2, pfcrt, and arps10 genes not reported in a study by Miotto et al.,(8) and other previous studies in Africa (29) were detected in our current study. There is need to validate the role the identified background mutations play in artemisinin resistance development among African P. falciparum parasites.”

**Response:** This is noted and has been effected in the revised manuscript.

**Comment:** Lines 299-302: In the following sentences it is not clear what is actually “similar”: “A recent study in South Sudan (26) which analyzed samples collected in 2015-2017 after introduction of artemisinin agents in malaria treatment detected genetic background mutation in Pfcrt N326S gene which was previously reported in Southeast Asia and associated with artemisinin resistance. This is similar to a finding from our current study where we detected a background mutation, V127M in the arps10 gene that has been shown to support development of K13 mutations associated with artemisinin resistance in Southeast Asia(8)”
Add the following “While we did not find Pfcrt N326S SNP in our samples, we detected…”
“… after introduction of artemisinin agents in malaria treatment detected genetic background mutation in Pfcrt N326S gene which was previously reported in Southeast Asia and associated with artemisinin resistance. While we did not find Pfcrt N326S SNP in our samples, we detected a background mutation, V127M in the arps10 gene that has been also shown to support development of K13 mutations associated with artemisinin resistance in Southeast Asia(8)”.

**Response:** Thanks for the comment. This has been effected in the revised manuscript

**Comment:** Line 320: This statement is ambiguous: “We detected different K13 SNPs in P. falciparum samples collected both prior (2005) and after (2013) introduction of artemisinin combination therapies in malaria treatment in Uganda.
Suggest to change the sentence to indicate that K13 SNPs identified in the present study are different to those previously implicated in artemisinin resistance in Africa and SEA. For example, “In our samples collected in 2005 and 2013 we detected K13 SNPs different to those previously implicated in artemisinin resistance (ref). or "We detected different K13 SNPs in P. falciparum samples collected in 2005 compared to those collected in 2013 after introduction of artemisinin combination therapies in malaria treatment in Uganda".

**Response:** The comment is noted, we have made the suggested adjustments in the revised manuscript.

**Comment:** Line 322: Our findings are similar to a recent study in Uganda that reported K13 mutations in the P. falciparum 323 parasites (30)- Needs clarification similar in what way?

**Response:** Thanks for the comment, this sentence has be deleted from the revised manuscript.

**Comment:** Line 323-324. “Additionally, our study found a general increase in the prevalence of K13 SNPs in samples collected after (2013) introduction of ACTs in malaria treatment in Uganda”.
This statement is bit misleading since the is no statistically significant differences in the prevalences of these SNPs before and after introduction of ACTs.

**Response:** The statement has been corrected in the revised manuscript. Although not statistically significant, there is an observed trend towards increase in the prevalence of K13 mutations following introduction of artemisinin agents in malaria treatment.

**Comment:** The similar statement in Conclusions (below) can also be misinterpreted with the vagueness of the term “generally”. While it is correct in case of novel SNPs in pfmdr2, differences in SNPs for K13 and arsp10 were not statistically significant. Conclusions, should also reflect the findings related to the primary aim of the study and state that there were neither K13 SNPs previously implicated in artemisinin resistance in Africa and SEA, nor mutations in the associated background genes identified in this study with exception of one SNP in arps10 gene.

**Response:** This is noted and has been corrected in the revised manuscript

**Comment:** Line 331 “The proportions of K13, arsp10, and pfmdr-2 gene mutations were generally higher in P. falciparum parasites collected after introduction of artemisinin combination therapies in malaria treatment in Uganda

**Response:** This has been revised to refer to only pfmdr-2 gene mutation in the revised manuscript.

Other comments.

**Comment:** Line 34: Spaces are needed between P. and falciparum

**Response:** This has been corrected

**Comment:** Lines 36,56, 115: Saying it is “a decade” between 2005 and 2013 is not accurate-it is 8 years.

**Response:** Thanks for the comment, this has been corrected in the revised manuscript

**Comment:** Line 257 fcrt replace for pfcrt

**Response:** This has been corrected in the revised manuscript.
