# Supplementary material for: Prevalence of arps10, fd, pfmdr-2, pfcrt and pfkelch13 gene mutations in Plasmodium falciparum parasite population in Uganda
Source: PLoS One. 2022 May 5;17(5):e0268095. doi: 10.1371/journal.pone.0268095 (PMC9070901; doi:10.1371/journal.pone.0268095)
Supplement: Attachment — Submitted filename: RESPONSE TO REVIEWERs.doc [file pone.0268095.s008.doc]

**RESPONSE TO REVIEWER’S COMMENTS ON MANUSCRIPT PONE-D-21-33254R3**

**Comments, Reviewer #1**: Authors have addressed almost all of the comments. I believe that the MS can be accepted for publication after the following minor comments are addressed-please see below. The version with Track changes is used for line references.

**Response:** Thanks for the comments that have helped improve the manuscript, we are grateful.

**Comment, Reviewer #1:** Line 47: Authors inserted “…and arps10 (p=0.238)”, which is repetitive with the same statement below in lines 56-57 “…There was no statistically significant difference relationship (p=0.238) in the prevalence of arps10 SNPs..”. Suggest to delete “and arps10 (p=0.238)” in line 47.

**Response:** This has been effected in the revised manuscript

**Comment, Reviewer #1:** Line 250: Table 2 in Column “Prevalence of mutation, %(n/N)” “N” is actually not shown, while it is stated in the column heading and in the footnote to the table. As I previously, suggested, N (the denominator) needs to be added for every number in this column consistent with the column’s heading. It would make it easier to comprehend the data and consistent with the format of data shown in the text.

**Response:** Thanks, this has been provided as guided in the revised manuscript

**Comment, Reviewer #1:** Line 114: “South east Asia” please change for Southeast or South East Asia.

**Response:** Thanks, this has been corrected in the revised manuscript.

**Comment, Reviewer #1:** Line 141-142 “…Confirmation of the presence of P. falciparum parasites in the stored(?) blood samples was done by two techniques namely malaria rapid diagnostic test (mRDT) and microscopy”. Were RDT and microscopy done before samples were frozen or after samples were thawed after storage. While RDTs are possible to do on frozen samples I am not sure if the quality slides could be produced after the blood is lysed upon thawing. If authors could clarify this statement.

**Response:** Sorry, we did not make it clear enough. Microscopy was performed in the field before the blood samples were frozen by the primary studies. In our current study, we conducted *pfHRP-2* malaria rapid diagnosis (mRDT) to screen for frozen samples that had *P. falciparum* parasites. This has been re-written in the revised manuscript revised manuscript.

**Comment, Reviewer #1:** Line 205: “Correlation analysis was done using Fisher’s exact test to assess the relationship between mutations and year of sample collection” Suggest to change it to “.. using Fisher’s exact test to assess the differences in the prevalence of mutations in 2005 and 2013.”

**Response:** Thanks, this has been corrected in the revised manuscript.

**Comment, Reviewer #1:** Line 213 “…(4.2%, 6/142) K13 SNPs were detected (Table 2)”. Suggest to replace with “… SNPs were detected (Tables 1 and 2)” to reference the Tables in their order of appearance.

**Response:** Thanks for the comment, this has been corrected in the revised manuscript
